# Self-Help App for Depression in People With Intellectual Disabilities: A Randomized Clinical Trial

**DOI:** 10.1001/jamanetworkopen.2025.36364

**Published:** 2025-10-09

**Authors:** Swantje Borsutzky, Lina Hannah Paul, Lara Rolvien, Steffen Moritz

**Affiliations:** 1 Department of Psychiatry and Psychotherapy, University Medical Center Hamburg-Eppendorf, Hamburg, Germany

## Abstract

**Importance:**

Individuals with intellectual disabilities (IDs) are at increased risk for mental health problems, including depression. However, access to effective therapeutic interventions is often limited.

**Objective:**

To evaluate the feasibility, acceptance, and efficacy of a self-help smartphone app designed to reduce depressive symptoms and improve self-esteem and quality of life in individuals with IDs.

**Design, Setting, and Participants:**

In this 2-arm randomized clinical trial, adults with IDs and depressive symptoms were enrolled online in Germany between April 1 and August 10, 2023. Of the 135 individuals who accessed the survey, 99 met the eligibility criteria. Participants were recruited via social media, third parties (eg, care institutions), and workplaces. Data were collected at baseline and after the intervention. Statistical analyses included complete case and intention-to-treat (ITT) approaches.

**Intervention:**

Participants were randomly assigned (1:1) to either the intervention group using a smartphone app in easy-to-read German mainly on cognitive behavioral therapy or a waiting list control group. Participants in both groups continued to receive care as usual, which may include routine psychosocial support, daily structure provided by caregivers, and access to general health services.

**Main Outcomes and Measures:**

The primary outcome was a reduction in depressive symptoms, measured by the Glasgow Depression Scale for People With a Learning Disability. Secondary outcomes were improvements in self-esteem, quality of life, and participant satisfaction.

**Results:**

Among the 99 participants (mean [SD] age, 34.9 [12.6] years; 54 [54.5%] female), 92 completed the postintervention assessment. The intervention group showed a significant reduction in depressive symptoms compared with the control group in ITT analyses (*F*_1,97_ = 7.52; *P* = .007; η_p_^2^ = 0.072; medium effect size), as well as significant improvements in quality of life (*F*_1,97_ = 5.09; *P* = .03; η_p_^2^ = 0.050; small to medium effect size) and in self-esteem (*F*_1,97_ = 17.94; *P* < .001; η_p_^2^ = 0.156; large effect size). Complete case analyses yielded consistent results on most outcome measures. High satisfaction ratings were reported.

**Conclusions and Relevance:**

In this randomized clinical trial, a self-guided smartphone app demonstrated efficacy in reducing depressive symptoms and enhancing quality of life and self-esteem among individuals with IDs. The findings suggest that smartphone-based interventions can provide effective support for this underserved population.

**Trial Registration:**

German Clinical Trials Register Identifier: DRKS00030858

## Key Points

**Question:**

Can a self-help smartphone app reduce depressive symptoms among individuals with intellectual disabilities (IDs)?

**Findings:**

In this randomized clinical trial including 99 participants with IDs, the use of a self-help smartphone app for an intervention period of 4 weeks significantly reduced depressive symptoms compared with the waiting list control condition in intention-to-treat analyses.

**Meaning:**

Smartphone app–based self-help interventions can provide effective support to reduce depressive symptoms in individuals with IDs.

## Teaser

This randomized clinical trial evaluates whether a smartphone-based self-help application improves symptoms of depression among individuals with intellectual disabilities.

## Introduction

Intellectual disabilities (IDs) affect 1% to 3% of the global population.^1,2^ Individuals with IDs are significantly more likely to experience mental health disorders than the general population, with approximately 34% affected,^3,4^ most commonly by clinical depression, followed by anxiety and psychotic disorders.^4,5,6^ Diagnostic overshadowing (misattribution of psychological symptoms to IDs) and reliance on self-report contribute to suspected underdiagnosis.^5,7,8^

While cognitive behavioral therapy (CBT) is recommended by national guidelines and shows promising results for patients with IDs and psychiatric disorders, effective implementation remains limited.^9,10,11,12^ Mental health services for this population are significantly underprovided due to systemic factors, including limited treatment access,^13,14,15,16^ lack of specialized psychotherapeutic and psychiatric services,^17,18^ low financial incentives for specialized mental health care,^19,20,21^ and patient-related factors such as dependence on caregivers,^22,23^ communication difficulties, and mobility limitations.^24,25,26^ Clinician-related barriers include lack of knowledge about effectiveness,^27^ diagnostic overshadowing,^16,25,28^ lack of expertise,^14,27,29,30^ and issues related to working with caregivers, such as lack of communication and transparency.^24^ This highlights the urgent need for accessible treatment methods.

Smartphone-based interventions are increasingly being explored for various mental health conditions and may help overcome implementation barriers in people with IDs by offering flexible, low-threshold, and cost-effective access. They can be adapted to specific needs—through adjustable text size, audio support, and screen readers—and have shown significant efficacy in reducing symptoms of depression, anxiety, stress, and eating disorders in individuals without IDs.^31,32,33^ However, their effectiveness in people with IDs with comorbid mental health conditions remains largely unknown.^34,35^

As part of the current study, we developed a self-help smartphone app named Happy (Glücklich in German).^36^ The app is freely available and was designed to be easily accessible, aiming to improve the mental well-being of those who might otherwise face barriers to traditional face-to-face therapy. The app is written in easy-to-read German, following European standards for making information easy to read and understand.^37^ These guidelines define a standardized linguistic format that includes short sentences, simple and familiar vocabulary, and a clear layout. The app aims to reduce depressive symptoms and improve self-esteem through short exercises in individuals with IDs and was developed based on the COGITO app,^38^ a transdiagnostic smartphone app whose effectiveness has been demonstrated in several randomized clinical trials (RCTs).^39,40,41,42^ The goal of the present study was to evaluate whether the smartphone-based study intervention would be feasible for and accepted by the target group and assess its ability to reduce depressive symptoms and improve self-esteem and quality of life, making it one of the few RCTs to address this particular target group in the field of electronic mental health.^34,35,43^

## Methods

### Study Design and Participants

The study protocol for this RCT was reviewed and approved by the Ethics Committee of the University Medical Center Hamburg-Eppendorf. All procedures were conducted in accordance with the Declaration of Helsinki.^44^ Participants were recruited online throughout Germany, and informed consent was obtained in written form from all participants or their legal representatives prior to participation. The study followed the Consolidated Standards of Reporting Trials (CONSORT) reporting guideline. The full study protocol is available in Supplement 1.

Participants aged 18 to 75 years were enrolled between April 1 and August 10, 2023, based on the following inclusion criteria: (1) self- or caregiver-reported IDs, (2) presence of depressive symptoms, (3) access to a smartphone and the internet, and (4) no acute suicidality or history of bipolar disorder, psychotic disorder, or alcohol dependence. The presence of IDs was not formally verified. We relied on recruitment through networks and institutions (eg, residential facilities, sheltered workshops) specializing in supporting individuals with diagnosed IDs. This approach ensured feasibility and ecological validity in this population.

A screening item documented whether the survey was completed by the participant or a caregiver. All study materials, including the app and questionnaires, were presented in easy-to-read German^37^ and supplemented with audio options, instructive images, and telephone support to improve accessibility across a range of cognitive abilities.

Depressive symptoms were assessed using the Glasgow Depression Scale for People With a Learning Disability (GDS-LD).^45^ While no minimum symptom score was required, all participants reported at least some depressive symptoms. This inclusive approach was intended to reflect clinical diversity in symptom severity, including subclinical presentations. Exclusion criteria were assessed via structured self- or caregiver-reported items during the baseline survey. Participants were encouraged to consult mental health professionals if uncertain.

### Recruitment

We used both direct and indirect methods for recruitment. The direct recruitment strategy targeted the population through social media platforms. Indirect recruitment was conducted via third parties, such as the German organization Lebenshilfe (Life Support Hamburg), residential groups, workplaces, psychotherapists, and researchers in the field. Additionally, we aimed to recruit participants via their social environment, including friends, peers, family members, and caregivers, by encouraging informal sharing of recruitment material. To support both pathways, we created an information sheet outlining the study’s scope and content in easy-to-read^37^ and standard language, shared via email and online. Participants were offered the financial incentive of a €20 voucher for survey completion. Most participants were recruited through friends and acquaintances (n = 29), indicating that information was informally shared within the social environment of the participants. This was followed by recruitment via cooperation with our local organization (n = 22), residential groups (n = 19), and workplaces (n = 14). Social media platforms yielded only 5 participants, while 1 participant was referred by a caregiver and another by a school. The remaining participants did not report their recruitment source.

### Randomization

Participants were randomized using a 1:1 allocation ratio into the intervention group or the waiting list control group. Randomization was conducted after the baseline assessment to ensure participants completed the initial measures prior to group assignment, minimizing potential bias. The randomization process was automated using the randomization function within the Qualtrics survey software, 2023 version. This approach guaranteed allocation concealment, as neither participants nor researchers were aware of group assignments until the randomization was completed. Both groups received identical instructions at the start of the study, with the intervention group gaining immediate access to the study app through an automated procedure integrated into the survey platform. After randomization, participants in the intervention group were directed to a landing page that displayed download instructions, including QR codes and download links. Additionally, the survey platform automatically sent emails with a detailed description of the download to the participants in the intervention group. All participants, regardless of group assignment, had access to care as usual (which may include routine psychosocial support, daily structure provided by caregivers, and access to general health services) throughout the study period. The intervention group received the study app in addition to care as usual, while the control group continued care as usual alone and received access to the app after the postintervention assessment.

### Data Collection and Assessment Points

Data were collected at baseline and again 4 weeks later. At baseline, sociodemographic data and psychiatric history were collected; depressive symptoms, self-esteem, and quality of life were measured at baseline and at the postintervention collection time. Additionally, at the postintervention collection time, participant satisfaction with the intervention was evaluated. All assessments were conducted online using Qualtrics. To ensure data consistency across time points, participants created a unique code name for themselves at baseline.

### Adaptations for the Target Group

At the beginning of the survey, all of which was presented in easy-to-read language,^37^ an introduction explained the operation of this study app as well as the procedures of the study. Various informational texts were provided throughout to enhance participants’ understanding. For longer texts, participants could listen to the content via audio files. Additionally, a telephone assistance service was available. Following the introduction, an initial item identified whether the respondent was the intended app user or a caregiver. Based on the response, participants were routed to the caregiver version or the self-report version. While this item did not formally assess cognitive abilities, those who navigated to and responded to this question demonstrated sufficient functional skills to complete the survey independently. The caregiver version matched the self-report questionnaires but used third-person phrasing. Validated questionnaires were adapted to an online format. To enhance user engagement, smiley face icons were incorporated into the rating scales. Inversely worded questions were rephrased to improve comprehensibility, and additional explanations or examples were included for the same purpose. The online survey was accessible via computer, tablet, or smartphone, with a mean completion time of approximately 15 minutes. All materials^37^ were reviewed by experts with the German organization Lebenshilfe to ensure the texts met the official standards of Inclusion Europe.

### Outcome Measures

To examine temporal score stability, we calculated preintervention-postintervention correlations for each questionnaire (*r* range, 0.68-0.79). The 4-week interval matched the full intervention period to align with the structure of the study and to minimize participant burden. Given the cognitive abilities of the target group and to prevent fatigue and repetition-related confusion, additional questionnaires at shorter intervals were not used. The resulting coefficients should therefore be interpreted not as classic test-retest reliability estimates but rather as indicators of score consistency during a practically relevant period.

### Primary Outcome

The primary outcome measure was the GDS-LD,^45^ which was used to evaluate depressive symptoms at both time points. The GDS-LD demonstrates robust psychometric properties, including high internal consistency (Cronbach α = 0.90) and excellent test-retest reliability (*r* = 0.97).^46^ Additionally, it shows high sensitivity (96%) and specificity (90%) for detecting depression,^46^ as well as good convergent validity with other established depression scales.^47,48^

### Secondary Outcomes

Quality of life was assessed with 8 items we selected for this study from the World Health Organization Quality of Life Assessment–BREF (WHOQOL-BREF)^49^ in easy-to-read language.^37^ We used 1 item for overall quality of life, two items each for physical and mental health, 1 item for social relationships, and 2 items for environmental quality of life. To enhance accessibility, the WHOQOL-BREF was adapted using established easy-to-read principles.^37^ The adapted version retained item structure and scoring logic. Internal consistency of the adapted instrument was Cronbach α = 0.86, indicating high internal consistency.

The Rosenberg Self-Esteem Scale (RSE) was used to measure global self-esteem.^50^ We used the version adapted for people with IDs,^51^ which has reported internal consistency ranging from questionable to good (Cronbach α range, 0.62-0.82).^51,52,53^ The RSE shows large construct validity, with factorial analyses confirming a bidimensional structure and significant negative correlations with depressive symptoms and negative thinking.^54^

Patient satisfaction with the intervention was assessed using the 8-item Client Satisfaction Questionnaire (CSQ-8).^55^ This questionnaire is a reliable and valid tool for measuring client satisfaction across various health care settings. It exhibits high internal consistency (Cronbach α ≤0.97)^56,57^ and strong concurrent validity, with significant correlations with other satisfaction measures such as the Treatment Perceptions Questionnaire^58^ and the Recovery Self-Assessment.^57^ The scale’s single-factor structure is well supported, and it has been validated across multiple languages and cultural contexts.^56,59,60^ Its ability to be adapted to different cultural settings and its sensitivity to changes over time make it suitable for translation into easy-to-read language^37^ for individuals with IDs.

### Intervention

Participants in the intervention group used the study app for 4 weeks. The app suggested 1 new exercise daily to encourage engagement. Reminders were included to support satisfaction, adherence, and engagement.^45,46,47^ Because the app is intended for people with IDs, it uses easy-to-read language,^37^ colorful illustrations, and audio features so that it is easily accessible. The app targets mental well-being—especially depressive mood and low self-esteem—and is based on CBT^61^ and its third-wave approaches^62,63,64^, with metacognitive training components.^65^ It offers 37 practical exercises covering CBT core principles (eg, psychoeducation, behavioral activation, cognitive restructuring, resource activation, emotional awareness), but it does not yet include techniques such as exposure therapy or Socratic dialogue. Metacognitive elements encourage the users to observe and reflect on their thoughts and feelings. Exercises are nonsequential and can be freely selected, and a gamified reward system (color-coded stars) fosters motivation. Users can mark favorites for repetition.

The study app is based on our research group‘s COGITO app,^38^ which includes 230 brief exercises across multiple domains (eg, mood and self-esteem, mindfulness and inner peace) and is available in 17 languages. Both apps share core features such as a reward system and reminders and function as open-access, nonmedical self-help tools.

### Control Condition

Participants in the waiting list control group received care as usual, if any. They gained access to the study app after completing the postintervention assessment.

### Feasibility Assessment

Feasibility was assessed via (1) study adherence, (2) app use, and (3) app satisfaction (using the adapted CSQ-8). At the postintervention assessment, participants in the intervention group self-reported frequency of app use with predefined categories (eg, daily, 3-4 times per week, 1-2 times per week, <1 time per week). No back-end use data were collected.

### Statistical Analyses

Analyses were conducted using SPSS Statistics, version 29.0.1 (IBM Corporation). Both intention-to-treat (ITT) and complete case analyses were performed. Baseline differences were compared using paired-sample *t* tests. Outcomes (depression scores, self-esteem, and quality of life) were analyzed via 2-way analyses of variance with time (within participant) and condition (intervention group vs control group; between participants). A 2-tailed α level of *P* < .05 was applied. A priori power analysis indicated that 99 participants would be sufficient to detect a between-group difference at a small to medium effect size (Cohen *d* = 0.35).

## Results

As shown in the Figure, 135 participants accessed the survey; 36 were excluded (18 due to discontinuation, 18 due to ineligibility). The remaining 99 participants were randomized into the intervention group (n = 50) and the waiting list control group (n = 49). Seven participants dropped out after completing the survey (3 in the intervention group and 4 in the control group), with no difference across conditions (χ^2^_1_ = 0.133; *P* = .72). Retention during the intervention period was high, with 93 of 99 participants (94.0%) completing the postintervention assessment. Adherence was also strong, with 42 of 46 participants (91.3%) providing complete data about app usage.

**Figure.**
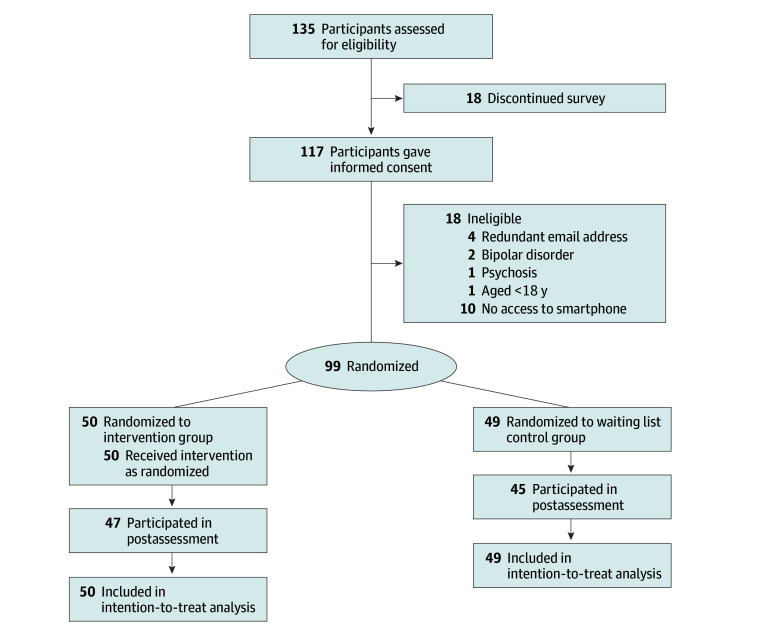
Participant Flowchart

### Sample Description

Table 1 presents baseline demographic characteristics of the participants. The mean (SD) age was 34.9 (12.6) years; 54 participants (54.5%) were female and 45 (45.5%) were male. Most assessments were completed by the participants themselves (88 [88.9%]); 11 caregivers (11.1%) filled out the assessments. No significant baseline differences were found for depression (GDS-LD, *t*_97_ = 1.19; *P* = .24) or quality of life (WHO-QOL, *t*_97_ = 1.38; *P* = .17); self-esteem was higher in the intervention group (RSE, *t*_97_ = 2.35; *P* = .02). Adjusted analyses of covariance confirmed that significant group differences remained (*F*_2, 96_ = 16.67; *P* < .001).

**Table 1.** Baseline Demographic Characteristics of Participants

Characteristic	Study group, No. (%)	
	Intervention group (n = 50)	Waiting list control group (n = 49)
Age, mean (SD), y	35.5 (11.1)	34.2 (11.2)
Sex		
Male	23 (46.0)	22 (44.9)
Female	27 (54.0)	27 (55.1)
Educational attainment		
Elementary school	0	1 (2.0)
Lower secondary school	11 (22.0)	12 (24.5)
Intermediate secondary school	14 (28.0)	16 (32.7)
Academic secondary school	3 (6.0)	5 (10.2)
Special school	22 (42.0)	15 (30.6)
Living situation		
Lives alone and receives no help	13 (26.0)	17 (34.7)
Lives alone with help from friends or family	4 (8.0)	4 (8.2)
Lives alone with help from caregiver or care service	7 (14.0)	3 (6.1)
Lives with parents	9 (18.0)	7 (14.3)
Lives in a residential group and receives help from staff	13 (26.0)	17 (34.7)
Lives in a nursing home and receives help from staff	2 (4.0)	1 (2.0)
Other	2 (4.0)	0
Work		
Goes to school	0	2 (4.1)
Works in a sheltered workshop	16 (32.0)	11 (22.4)
Works elsewhere	12 (24.0)	20 (40.8)
Is doing an apprenticeship	10 (20.0)	6 (12.2)
Does not work; receives welfare benefits or Citizens’ Basic Income	0	2 (4.1)
Is retired	4 (8.0)	1 (0.2)
Works in a day habilitation center	8 (14.0)	7 (14.3)

### Intervention Use

App usage in the intervention group was high; 13 (28.3%) used it 5 to 7 times per week, 19 (41.3%) used it 3 to 4 times per week, and 10 (21.7%) used it 1 to 2 times per week. Only 4 participants (8.7%) used the study app less than once per week. All participants who reported app usage data (n = 46) reported using the app at least once during the intervention period.

### Between-Group Differences

Rater type (caregiver vs patient) had no significant interaction effects and was removed from the final models. Table 2 summarizes all results. The ITT (last-observation-carried forward) showed significantly greater reduction in depressive symptoms in the intervention group (primary outcome: *F*_1,97_ = 7.52; η_p_^2^ = 0.072 [medium effect]) (*P* = .007) compared with the control group, along with significant improvement in quality of life (*F*_1,97_ = 5.09; η_p_^2^ = 0.050 [small to medium effect]) (*P* = .03) and self-esteem (*F*_1,97_ = 17.94; η_p_^2^ = 0.156 [large effect]) (*P* < .001).

**Table 2.** Within-Group and Between-Group 2-Way Analysis of Variance Results for Primary and Secondary Outcomes

Measure	Study group^a^						Complete case analysis^b^^,^^c^			ITT analysis^b^^,^^d^		
	Intervention group (n = 50)			Waiting list control group (n = 49)								
	Mean (SD)		Cohen *d* (95% CI)	Mean (SD)		Cohen *d* (95% CI)	*F*_1,90_	*P* value	η_p_^2^	*F*_1,97_	*P* value	η_p_^2^
	Preintervention	Postintervention		Preintervention	Postintervention							
GDS-LD^e^	7.86 (6.94)	5.72 (5.69)	.437 (0.144 to 0.725)	9.61 (6.07)	10.27 (6.34)	−.129 (−0.409 to 0.153)	5.47	.02	0.057	7.52	.007	0.072
WHOQOL-BREF^f^	28.04 (4.06)	31.08 (3.74)	−.952 (−1.283 to −0.613)	27.20 (3.58)	28.67 (4.27)	−.452 (−0.744 to −0.155)	3.92	.05	0.042	5.09	.03	0.050
RSE^g^	23.24 (3.33)	23.78 (2.84)	−.174 (−0.452 to −0.106)	21.71 (2.62)	20.96 (2.62)	.371 (0.079 to 0.659)	16.04	<.001	0.151	17.94	<.001	0.156

Abbreviations: GDS-LD, Glasgow Depression Scale for People With a Learning Disability; RSE, Rosenberg Self-Esteem Scale; WHOQOL-BREF, World Health Organization Quality of Life.

^a^ Unadjusted (raw) means and SDs for preintervention and postintervention outcomes are based on an intention-to-treat (ITT) sample using last observation carried forward (LOCF). Within-group effect sizes (Cohen *d*) with 95% CIs are reported.

^b^ Results of 2-way analysis of variance comparing between-group differences over time (group × time interaction) are shown.

^c^ Includes 47 participants in the intervention group and 45 in the waiting list control group.

^d^ Using LOCF; includes 50 participants in the intervention group and 49 in the waiting list control group.

^e^ Scores range from 0 to 26, with higher scores indicating greater levels of depressive symptoms.

^f^ Scores range from 26 to 40, with higher scores indicating better perceived quality of life.

^g^ Scores range from 10 to 40, with higher scores indicating greater self-esteem.

The complete case analyses yielded comparable results: greater reduction in intervention group vs control group depression scores over time (*F*_1,90_ = 5.47; η_p_^2^ = 0.057 [small to medium effect]) (*P* = .02). For the secondary outcome quality of life, the between-group effect reached a small to medium effect size but failed to reach significance (*F*_1,90_ = 3.92; η_p_^2^ = 0.042) (*P* = .05). The secondary outcome self-esteem showed a significantly greater increase in the intervention group compared with the control group (*F*_1,90_ = 16.04; η_p_^2^ = 0.151 [large effect]) (*P* < .001). Notably, these group differences were primarily driven by deterioration of symptoms in the control group rather than significant improvement in the intervention group.

### CSQ-8 and Subjective Appraisal

The study app was appraised positively by users according to the CSQ-8 (excellent or good ratings given by at least 39 users [84.8%] in all items) (Table 3). For example, 45 users (97.8%) rated the quality of the app positively, 43 (93.5%) would recommend the app to a friend, and 41 (89.1%) would use it again. Test-retest reliability (completers: baseline vs 4 weeks later) was satisfactory for the GDS-LD (*r* = 0.64), the WHOQOL-BREF (*r* = 0. 62), and the RSE (*r* = 0.66) (*P* < .001).

**Table 3.** Subjective Appraisal of Participants Who Used the Study App

CSQ-8 item	Mean (SD) score [No. (%) of positive respondents]^a^		
	Filled out by caregiver (n = 9)	Filled out by participant (n = 37)	Total (n = 46)
How do you rate the quality of [the app]? (not good [1] or less good [2] vs good [3] or excellent [4])	3.33 (0.50) [9 (100)]	3.41 (0.56) [36 (97.3)]	3.39 (0.54) [45 (97.8)]
Did you receive the type of treatment you expected to receive? (not at all [1] or not really [2] vs in general yes [3] or yes, absolutely [4])	3.22 (0.44) [9 (100)]	3.38 (0.64) [34 (91.9)]	3.35 (0.60) [43 (93.5)]
To what extent did [the app] meet your needs? (it did not meet my needs [1] or it met few of my needs [2] vs it met most of my needs [3] or it met all of my needs [4])	3.44 (0.53) [9 (100)]	3.24 (0.69) [34 (91.9)]	3.28 (0.66) [43 (93.5)]
Would you recommend [the app] to a friend with similar symptoms? (definitely not [1] or probably not [2] vs probably yes [3] of absolutely [4])	3.22 (0.44) [9 (100)]	3.43 (0.65) [34 (91.9)]	3.39 (0.61) [43 (93.5)]
How happy are you about the extent of the help you have received through using [the app]? (dissatisfied [1] or somewhat dissatisfied [2] vs mostly satisfied [3] or very satisfied [4])	3.56 (0.53) [9 (100)]	3.43 (0.55) [36 (97.3)]	3.46 (0.57) [45 (97.8)]
Did [the app] help you cope with your problems more successfully? (no, it did not help me at all [1] or no, it did not help me that much [2] vs yes, it helped me a little [3] or yes, it absolutely helped me [4])	3.33 (0.50) [9 (100)]	3.35 (0.59) [35 (94.6)]	3.35 (0.56) [44 (95.7)]
How satisfied are you with [the app] in general? (unsatisfied [1] or somewhat unsatisfied [2] vs mostly satisfied [3] or very satisfied [4])	3.33 (0.50) [9 (100)]	3.51 (0.56) [36 (97.3)]	3.48 (0.55) [45 (97.8)]
Would you use [the app] again? (definitely not [1] or probably not [2] vs probably yes [3] or yes [4])	3.11 (0.78) [7 (77.8)]	3.35 (0.63) [34 (91.9)]	3.30 (0.66) [41 (89.1)]
Has [the app] improved your mood? (definitely not [1] or probably not [2] vs probably yes [3] or yes [4])	3.11 (0.78) [7 (77.8)]	3.19 (0.74) [32 (86.5)]	3.17 (0.74) [39 (84.8)]

^a^ Responses include the 2 positive response options; higher ratings on the Likert scale correlate with increased participant satisfaction.

## Discussion

This is the first RCT, to our knowledge, to evaluate a smartphone-based self-help app for depressive symptoms in individuals with IDs. The results address an important research gap and suggest that the study app offers promising and accessible mental health intervention for this underserved population.

Compared with the waiting list control participants, app users showed reduced depressive symptoms (primary outcome, medium effect), improved quality of life (small to medium effect), and stabilized self-esteem (large effect), although the latter was primarily due to deterioration of symptoms in the waiting list control group. The app received positive evaluations: 97.8% rated its quality as good and were satisfied with the support received, and 89.1% said that they would use it again. These findings indicate high acceptability, user friendliness, and accessibility—key features in interventions targeting people with IDs. This is especially relevant given that satisfaction is linked to adherence and long-term use in digital interventions for physical and mental conditions.^66,67,68^ The self-reported high frequency of app use during the 4-week period supports the intervention’s feasibility and acceptability.

### Strengths and Limitations

Among its strengths, this study had a relatively large and heterogeneous sample for this underserved population and achieved high retention (94.0%) and adherence (91.3%), exceeding typical engagement levels in similar interventions.^34,35,43^ This likely contributed to the observed improvements.^39,69^ All 3 questionnaires showed good test-retest reliability, ensuring consistency of data. Randomization was largely successful, with only self-esteem differing at baseline. The broad inclusion criteria allowed participation by individuals with mild depressive symptoms and various degrees of IDs, resulting in a more diverse, representative sample and enhancing generalizability.

The study findings suggest that online research with individuals with IDs is feasible. A high follow-up rate at 4 weeks (94.0%) was achieved through strategies such as incentives, follow-up reminders, caregiver support, low-barrier access (eg, audio files, phone support), flexible data collection (online surveys), and close collaboration with Life Support Hamburg. Barrier reduction likely played a key role.^70^ Together, these strengths enhance the study’s validity and highlight a research design suitable for working with underserved populations.

This study also has several limitations. First, outcomes were collected via self-report or proxy report, which may introduce bias, particularly social desirability.^71^ However, the risk was mitigated by the participants’ functional level and the involvement of caregivers.^72^ Second, although computer-based randomization was used, the separate allocation paths (caregiver, self) may have introduced minor bias. Additionally, individual differences in self-esteem may not have been fully accounted for due to the heterogeneity of the sample.^73^ Third, ID status was based on self-report or caregiver report and recruitment through specialized networks. While this reflects clinical settings and facilitates participation, it may reduce diagnostic precision and limit generalizability. Regardless, the results were consistent across self-report and proxy reports. Fourth, while the waiting list design minimized burden and was ethically acceptable, it limits conclusions regarding mechanisms of change. Active control conditions would improve internal validity. Fifth, although the WHOQOL-BREF was adapted for cognitive accessibility and showed good internal consistency, further adaptation of the easy-to-read^37^ version with visual Likert scales is recommended. Sixth, app use was self-reported rather than objectively tracked; although this approach allowed a scalable assessment, objective use data would be more accurate. Finally, the short intervention period and lack of long-term follow-up limited insight regarding sustained effects.

## Conclusions

In this RCT, use of the study app significantly reduced depressive symptoms and improved quality of life and self-esteem among individuals with IDs. High rates of use and satisfaction indicate that the app is accessible, engaging, and tailored to this target group. Future studies should incorporate larger sample sizes and active control groups. Longer intervention periods and a follow-up assessment are needed to determine sustained benefits. Future research should also investigate moderators of effectiveness (eg, symptom severity, cognitive functioning) and determine who the app works best for. Objective usage data should be collected to link engagement with outcomes.

Clarifying the optimal use of the intervention—whether as stand-alone, bridge, preventive, or maintenance support—will be essential for routine application. Clinical implementation studies should explore caregiver involvement, remaining accessibility barriers, and use outside research contexts. To reduce participation barriers in future studies, simplified language, visuals, and audio support should be standard. Support from caregivers or research staff and manageable survey length can further enhance participation. Preliminary field testing with the target group can help identify and address additional barriers.

This study contributes important evidence to address the treatment gap for individuals with IDs. The app may serve as interim support for those awaiting regular therapy or those with mild symptoms, or for preventive use. Given the increasing smartphone access in this group, app-based interventions represent a promising strategy to improve access to mental health care.

## Notes

Supplement 1.
Trial Protocol

Supplement 2.
Data Sharing Statement
