# Supplementary material for: Self-Help App for Depression in People With Intellectual Disabilities: A Randomized Clinical Trial
Source: JAMA Netw Open. 2025 Oct 9;8(10):e2536364. doi: 10.1001/jamanetworkopen.2025.36364 (PMC12511992; doi:10.1001/jamanetworkopen.2025.36364)
Supplement: Supplement 1. — Trial Protocol [file jamanetwopen-e2536364-s001.pdf]

Director of Studies

Prof. Dr. Steffen Moritz

Königsberger Street 5

25462 Hamburg

Institute

Clinic and Polyclinics for Psychiatry and Psychotherapy

Cooperation partner

no cooperation partners

Funding

Own funds

Planned duration

01.11.2022 until 01.04.2023

Background: Intellectual impairment according to DSM-V is defined as a developmental disorder of intellectual and adaptive skills (American Psychiatric Association, 2013). Worldwide, approx. 1 - 3 % of the population live with such an impairment (Maulik et al., 2011; McBride et al., 2021; Platt et al., 2019a; Schanze, 2014). In Germany, with a population of around 83 million people, this would affect around 830,00 to 2.5 million people (as of 2021; Federal Statistical Office, 2022b). They are more likely to have one or more physical and/or mental illnesses than people without intellectual disabilities (Cooper et al., 2015; Mazza et al., 2020). The prevalence of mental illness varies greatly depending on the study and is between 11% and 65% (Bratek et al., 2017; Platt et al., 2019b; Schützwohl et al., 2016; Sheehan et al., 2015). A meta-analysis from 2020 summarizes the results of 22 studies such that the prevalence of comorbid mental illnesses is around 34% (Mazza et al., 2020) and is therefore higher than the prevalence of mental illnesses in the average German population (Jacobi et al., 2014). The most common mental illness in many studies was depression (Mazza et al., 2020; Peña-Salazar et al., 2020; Sheehan et al., 2015). However, at between 6% and 14%, the prevalence of depression was not higher than in the general population (Maïano et al., 2018; Platt et al., 2019b; Scheirs et al., 2022; Steffen et al., 2020). However, it is questionable whether the stated prevalences represent a realistic picture, as many studies assume a not insignificant underdiagnosis (Peña-Salazar et al., 2020; Scheirs et al., 2022). The reasons for this are that other symptoms are often in the foreground in cases of severe intellectual impairment (diagnostic overshadowing). In addition, diagnosis is more difficult, as it is sometimes not possible to provide information independently (Boat & Wu, 2015). In addition to the diagnostic hurdles, people with intellectual disabilities usually fall ill earlier (Munir, 2016) and are more frequently

affected by a chronic course of depression (Cooper et al., 2007; Richards et al., 2001). Although psychotherapeutic methods, in particular cognitive behavioral therapy, have proven to be effective in this target group, it can be assumed that there is a severe underuse in the inpatient and outpatient sector (Evans et al., 2012; Lipinski et al., 2019; Whittle et al., 2017). The reasons for the lack of care can be traced back to the care system (low financial incentives, lack of accessibility, general lack of care), those affected (dependence on caregivers, mobility restrictions), but also practitioners (fear of contact, doubts about effectiveness, lack of expertise, additional effort due to e.g. involving caregivers). In order to overcome these barriers, low-threshold support services are needed that can address the aforementioned treatment barriers and reach more people affected. One possible alternative to conventional face-to-face psychotherapy is smartphone-based interventions, as they are flexible, low-threshold, resource-friendly and cost-effective. According to a meta-analysis, there is evidence for the effectiveness of smartphone interventions in reducing depressive symptoms in the average population, which is in the small effect size range g = 0.22 (Firth et al., 2017). To date, we are not aware of any comparable interventions that address the psychological symptoms of people with intellectual disabilities. For these reasons, we have developed a smartphone app that is tailored to their specific needs. The app is written in simple language, is easy to use and is designed to reduce depressive symptoms and improve self-esteem with short exercises. The Glücklich app was developed on the basis of the COGITO app. The COGITO app is a transdiagnostic smartphone app whose effectiveness has already been proven in two RCTs (Lüdtcke et al., 2018; Bruhns et al., 2021). The Glücklich app is based on cognitive behavioral therapy methods (CBT) and third wave methods (e.g. acceptance, mindfulness). The exercises are text-based, but can also be read aloud as an audio file if required (e.g. visual impairment, reading difficulties). Drawings that reproduce the content of the exercises provide support. To ensure that the exercises are used regularly in everyday life in order to increase adherence and effectiveness, users are reminded daily to perform the exercises via short push notifications.

Procedure: The planned pilot study is a randomized controlled trial with two conditions: an intervention group, which can use the self-help smartphone app over a period of 4 weeks, and a waiting control group, which only receives access to the self-help smartphone app after completion of the post-survey. In order to measure a change over time, two measurement points with detailed online diagnostics are planned: a baseline test before the start of the intervention period (T0) and a post-test after four weeks (T1). The questionnaires are collected online via Qualtrics® (the survey via Qualtrics is compliant with the General Data Protection Regulation, GDPR; personal data such as email addresses are permanently destroyed and no IP addresses are stored). The Glasgow Depression Scale (GDS) serves as the primary success parameter for assessing depressive symptoms. The GDS is a self-assessment questionnaire for mild to moderate intelligence reduction (IM). For respondents who are unable to self-rate, we

assess depressive symptoms using the Glasgow Depression Scale - Carer Supplement (GDS-CS). The GDS and GDS-CS show a high correlation of r=0.93 (Cuthil et al., 2003). A global item of the WHOQOL-BREF for measuring quality of life serves as a secondary success parameter. Instead of the 5-point response scale (“very poor”, “poor”, “fair”, “good”, “very good”), respondents can answer on a smiley analog scale. The Rosenberg Self-Esteem Scale (RSE) is used to measure self-esteem. Its validity and reliability were demonstrated in a sample group of people with intellectual disabilities (Park & Park, 2019). Secondary parameters should also be collected by means of a third-party rating if respondents are unable to complete the self-rating. Socio-demographic data is also collected at T0. At T1, subjective ratings and user-friendliness are also collected. In order to link the data collected at both measurement times, respondents create a code name at T0. Recruitment is supported by Lebenshilfe Hamburg, which is funding the project. This means that test persons can be contacted personally or at their facilities. In parallel, affected persons and/or their relatives/caregivers will be recruited via social media (e.g. Facebook campaign) and a flyer campaign. Inclusion and exclusion criteria are: intellectual impairment, informed consent, availability of internet access and a smartphone, willingness to take part in two pseudonymous online surveys, each lasting approx. 15 minutes each, willingness to take part in the 4-week self-help smartphone app, willingness to use the self-help smartphone app on your own responsibility, willingness to leave an anonymized email address for the survey, presence of depressive symptoms, no acute suicidal tendencies (recorded via an item of the PHQ-9) leads to exclusion) and no bipolar or psychotic illness currently or in the past (recorded with the question “Have you ever been diagnosed with bipolar disorder/mania or schizophrenia/psychosis? “) . In the participant information, it is pointed out that those affected who experience a worsening of symptoms can contact the advice hotline of the Federal Center for Health Education (BZgA). The relevant telephone number is also listed. In addition, caregivers are informed about participation in the study and instructed to provide support in the event of worsening symptoms and to stop participation in the study. In the event of exclusion due to suicidal tendencies, further emergency numbers are provided. It is expressly stated in the participant information that the program is not suitable for acute crises and cannot replace psychotherapy. To determine the effectiveness of the self-help smartphone app, both intention-to-treat (ITT) and per-protocol (PP) analyses are carried out. In the ITT analyses, missing values are replaced using multiple imputations (MI). The main analyses are calculated using analyses of covariance (ANCOVAs) with baseline values as covariates. Possible moderators are to be checked using regression analyses. A sample calculation was not carried out, as no comparable studies have been conducted to date. The aim of the pilot study is to recruit a sample of N = 50 in order to gain initial insights into the feasibility and effectiveness of app-based self-help for this target group.

Expected benefit: A greater reduction in depressive symptoms and a greater increase in the quality of life and self-esteem of the subjects in the intervention group compared to

the control group is expected over time. The long-term goal of the study is to improve the mental health of people with intellectual disabilities.
