# Supplementary material for: Self-Help App for Depression in People With Intellectual Disabilities: A Randomized Clinical Trial
Source: JAMA Netw Open. 2025 Oct 9;8(10):e2536364. doi: 10.1001/jamanetworkopen.2025.36364 (PMC12511992; doi:10.1001/jamanetworkopen.2025.36364)
Supplement: Supplement 2. — Data Sharing Statement [file jamanetwopen-e2536364-s002.pdf]

Data Sharing Statement

Borsutzky. Self-Help App for Depression in People With Intellectual Disabilities. *JAMA Netw Open*. Published October 09, 2025. doi:10.1001/jamanetworkopen.2025.36364

Data
Additional Information: DRKS00030858 https://drks.de/register/de/trial/DRKS00030858
Data available: Yes
Data types: Other (please specify)
Additional Information: Data will be made available upon request
How to access data: request must be sent to corresponding author
When available: With publication

Supporting Documents
Document types: Other (please specify)
Additional Information: Anonymized data will be made available upon reasonable request.
How to access documents: s.borsutzky@uke.de
When available: With publication

Additional Information
Who can access the data: Researchers whose proposed use of the data has been approved.
Types of analyses: Anonymized data will be made available upon reasonable request for replication and verification of results by researchers affiliated with academic institutions.
Mechanisms of data availability: Anonymized data will be made available upon reasonable request, following approval of a research proposal and with a signed data access agreement."
Any additional restrictions: The data may not be used for commercial purposes or redistributed without explicit permission.
